# Improved de-inking of inkjet-printed paper using environmentally friendly atmospheric pressure low temperature plasma for paper recycling

**DOI:** 10.1038/s41598-019-50495-4

**Published:** 2019-10-01

**Authors:** Rodolphe Mauchauffé, Seung Jun Lee, Isaac Han, Sang Hyeong Kim, Se Youn Moon

**Affiliations:** 1 Department of Quantum System Engineering, Chonbuk National University, 567 Baekje-daero, Deokjin-gu, Jeonju, Jeollabuk-do 54896 Republic of Korea; 2 Altoran Research Institute, 10-41, Bokyongbuk-ro 17, Yuseong-gu Daejeon-city, 34160 Republic of Korea

**Keywords:** Plasma physics, Environmental sciences

## Abstract

Nowadays, due to environmental pollution and natural energy consumption caused by waste paper, many researches are being conducted on the reuse of printed-paper. To recycle the paper, de-inking has to be performed. In this article, in order to reduce the use of the commonly used de-inking chemicals, the effect of an atmospheric pressure helium plasma treatment on the de-inking enhancement of printed-paper is studied. Through colorimeter and UV-visible spectrometer measurements the plasma treatment is shown to speed up the de-inking. While SEM observations and FTIR measurements suggest that the paper quality is retained upon plasma treatment, the increase of surface hydrophilicity measured by water contact angle measurements, compared to non-treated paper, is believed to enhance the fiber swelling of the paper and lead to a faster ink removal.

## Introduction

Paper industry is well known to consume enormous quantities of raw materials and energy. Paper recycling is more and more performed around the world to try to reduce the use of natural resources^1–3^. In order to recycle paper, de-inking has to be performed to improve the final color and overall quality of the recycled paper. Among the various de-inking methods, chemicals such as peroxides, caustic soda, and surfactants are commonly used^1,4^. The use of these chemicals leads to polluted water production and thus to costly waste water treatments^1–4^. Alternative methods to either reduce the use of these chemicals or to replace these compounds have to be found^2–4^. For example, bio-deinking via enzymatic treatment, employing cellulases, xylanases, pectinases or lipases, is one of these alternatives^4^. However, enzymes are extremely sensitive to the working conditions^4^. In addition, enzymes show limitations to remove hydrophilic inks such as inkjet and flexographic inks^1,4^. In order to provide a robust and easily up-scalable method to assist or enhance the current or newly developed de-inking processes, other ways have to be investigated. In this work, an atmospheric pressure helium plasma treatment-based method is studied. Open-air atmospheric plasma methods are drawing an ever growing attention to their ability to modify surfaces in one-step at low temperature without need of solvents and without high running cost vacuum systems, making them eco-friendly and easily up-scalable methods^5^. Indeed, open-air plasmas, such as dielectric barrier discharges, are able to modify surfaces either by the formation of functional coatings through the use of a film forming precursor in a discharge^6,7^ or by the direct modification of the materials surface through reactions with the reactive species present in discharges^8^. Atmospheric plasma treatments appear as a versatile solution to enhance the de-inking of paper.

Indeed, plasma treatments with gases such as helium, argon, oxygen, nitrogen or air are reported in the literature to lead to the increase of the surface hydrophilicity^9^. The paper wetting can play an important role in the swelling of the fibers and thus in the de-inking process efficiency^10–12^. Gaiolas *et al*. use a plasma at low pressure to treat paper in order to increase its hydrophilicity to minimize the disintegration time of the paper during the recycling process^10^. At atmospheric pressure, Pawlat *et al*. show the increase of hydrophilicity of the paper via treatment with a plasma jet^11^. Pykönen *et al*. study the atmospheric pressure plasma treatment of different types of paper with various plasma reactors and discuss the origin of the increase of surface energy upon plasma treatment^12^. To the best of our knowledge, no works on the effect of an atmospheric pressure plasma treatment on the de-inking of inkjet-printed paper are reported in the literature.

Herein, the effect of plasma treatment on the ink removal efficiency when treated samples are immersed in water is assessed. The de-inking efficiency is assessed by colorimeter and UV-visible spectrometer measurements. The morphology, chemistry and mechanical properties of treated paper are investigated through SEM imaging, water contact angle (WCA) measurement, Fourier transform infrared (FTIR) spectroscopy analyses and paper tensile testing. On the basis of these results, the de-inking mechanism is discussed.

## Materials and Methods

### Materials

A4 paper sheets (Double A, 80 g/m^2^, Korea) are printed using an inkjet printer (HP Photosmart 2575 All in-one) with HP95 (C8766WA) ink cartridge. Paper sheets are printed in red, blue and yellow.

### De-inking protocol and plasma treatment

**Figure 1:**
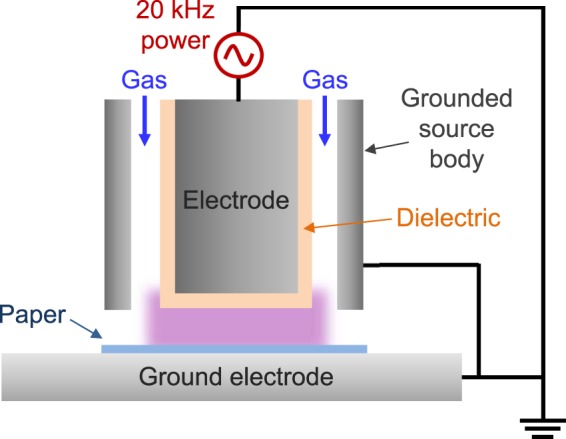
Schematic of the Dielectric Barrier Discharge (DBD) set-up.

Plasma treatment is performed using an atmospheric pressure dielectric barrier discharge as depicted in Fig. 1. The paper sample is fixed on an aluminum ground electrode. A helium plasma is ignited at 20 kHz, with a 1.5 kV voltage (about 16 W dissipated power) (Fig. S1). The helium flow rate is set to 4 L.min^−1^. The electrode consists in a cylindrical (2 cm diameter) metal electrode covered with alumina. The gap between the sample and the high voltage electrode is set to about 1 mm.

To investigate the effect of plasma on paper surfaces, printed papers are exposed to plasma and the plasma-treated areas are compared to non-exposed areas. The effect of plasma treatment time on de-inking is studied by exposing for 1, 2, 3, 4 and 5 minutes the printed papers to the plasma and subsequently soaking them in 200 mL of water for 1 minute under stirring at 80 RPM and then drying them at air. The effect of the water immersion time on de-inking is studied by soaking plasma-treated samples (3 minutes plasma treatment) in 200 mL of water for 1, 2, 5, 10 and 20 minutes under stirring at 80 RPM and then drying the samples at air. Since the efficiency of the de-inking could be influenced by the plasma self-heating and not by the effect of direct plasma exposure, the de-inking of printed papers plasma-treated or only heated with a heating plate is compared. The paper is heated for three minutes at 60 °C (similar to the temperature reached after 3 minutes plasma treatment) using a heating plate and soaked in water during 1 minute under stirring at 80 RPM. The reflectance is measured and compared to 3 minutes plasma-treated paper soaked in water during 1 minute.

### Paper characterization

To determine the extent of the de-inking, we measure the difference of color between the samples with a colorimeter (CR-20, KONICA MINOLTA). The colorimeter measures the *L*, *a*, and *b* values of the surfaces. The CIE *L* * *a* * *b* color space is commonly used to describe colors^13^. *L* corresponds to the brightness with *L* = 100 indicating white and *L* = 0 corresponding to black. The *a* value correspond to a scale ranging from green to red, with the highest value (positive value) corresponding to red and the lowest (negative value) to green. The *b* value corresponds to a blue-yellow gradient, with the highest value (positive value) corresponding to yellow and the lowest (negative value) to blue. Color measurements are performed both in the plasma-treated area and in the remote non-treated area. The changes in reflectance of the samples are also followed by UV-VIS spectroscopy (UV-2700, SHIMADZU). A digital analysis of the scanned samples after de-inking treatment is performed with the ImageJ software (NIH). For all these measurements, 4 cm × 8 cm samples printed in red, yellow and blue are used. Water contact angle (WCA) is measured using a contact angle measuring device (SDL200TEZD, FEMTOLAB). Fourier Transform Infrared Spectroscopy (FTIR) is performed with a ThermoFisher Scientific Nicolet iS 50 spectrometer using Attenuated Total Reflectance (ATR) mode. The paper surface is examined using a JEOL JSM-5900 scanning electron microscope. Tensile strength tests are performed using a 5982 Universal testing machine (Instron, USA).

## Results and Discussion

### Plasma de-inking of inkjet-printed paper

**Figure 2:**
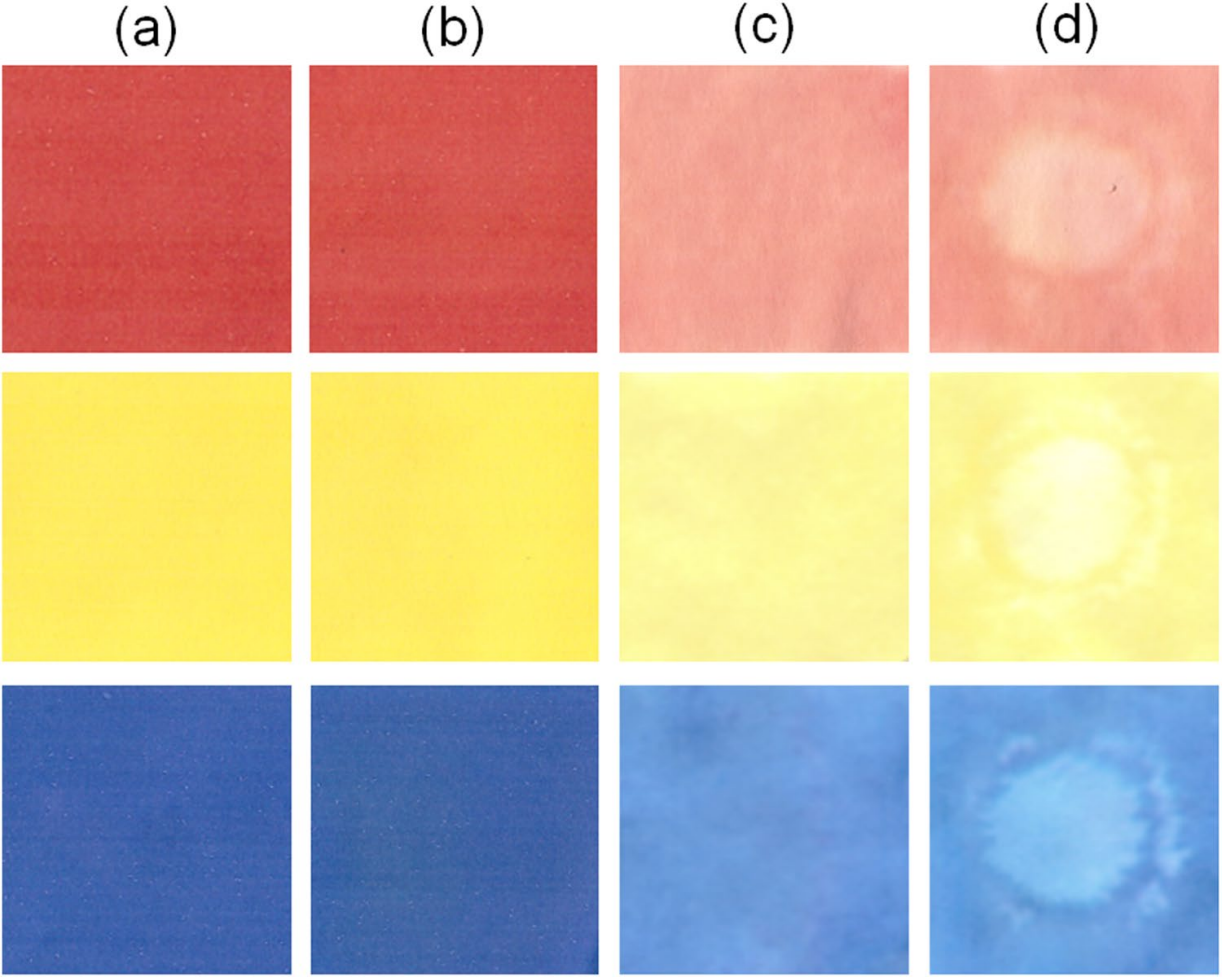
Scans of (**a**) original printed paper, (**b**) plasma treated printed paper, (**c**) non-treated area of printed paper soaked in water and (**d**) plasma-treated area of printed paper soaked in water.

**Figure 3:**
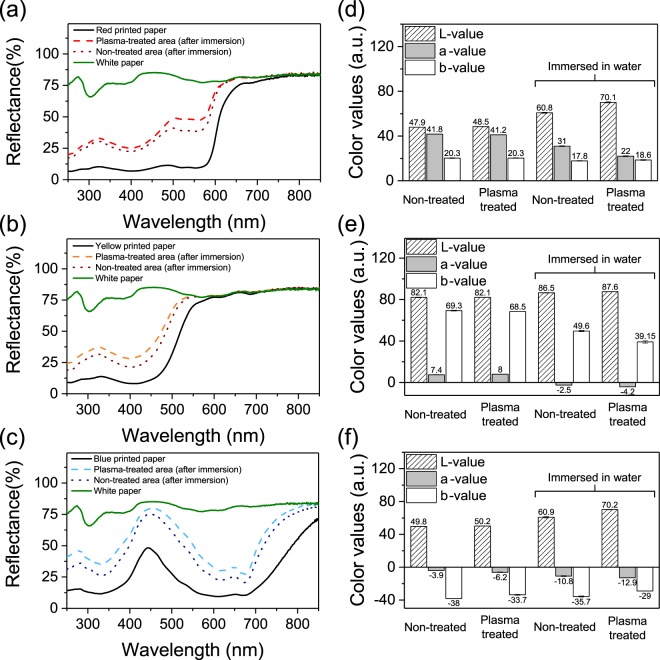
Reflectance measurements of (**a**) red printed paper, (**b**) yellow printed paper, (**c**) blue printed paper plasma treated (3 minutes) area and non-plasma-treated area after 1 minute immersion in water. The non-printed paper (white paper) and non-immersed non-treated printed paper spectra are reported on the figure for comparison. *L-*, *a-*, and *b-* values of (**d**) red, (**e**) yellow and (**f**) blue printed papers: non-treated and plasma treated areas color values are reported on the figure for both non-immersed papers and papers immersed 1 minute in water.

White papers (4 cm × 8 cm) printed in red, yellow and blue (Fig. 2a) are exposed to a plasma during 3 minutes (Fig. 2b). The samples are immersed in water, dried and scanned. As the plasma size is about 2 cm of diameter only a part of the paper is exposed to the plasma. Figure 2c,d are taken from the same printed samples after immersion in water. Figure 2c corresponds to the non-treated areas of the samples and Fig. 2d corresponds to the treated areas of the samples. Even though before and after plasma treatment (Fig. 2a,b), the samples seems to not differ much when viewed with the naked eye, upon immersion in water during 1 minute under stirring and subsequent drying at air, we can notice clear differences between not treated (Fig. 2c) or treated areas (Fig. 2d). Indeed, a difference in brightness is visible where the paper is directly exposed to the plasma (Fig. 2d), compared to the outside of the plasma treated area (Fig. 2d) or compared to the remote non-treated areas (Fig. 2c). In order to confirm these visual observations and assess the effect of the plasma on the de-inking, the samples are analyzed via reflectance measurements using a UV-Vis spectrometer (Fig. 3a–c) and the samples color values are measured with a colorimeter (Fig. 3d–f). Reflectance measurements performed with a UV-Vis spectrometer (Fig. 3a–c) shows that higher reflectance values are observed for all plasma-treated areas compared to non-treated areas after immersion in water, suggesting a higher ink removal.

A colorimeter giving the *L-a-b* color values is used to quantify the color changes both in plasma treated areas and non-treated areas. These measurements confirm that non-treated and plasma-treated areas, without immersion in water, have about the same color. However, as observed with the naked eye, after immersion in water, an increase of the *L* values (especially for red and blue papers) and a decrease of the *a* value for the red paper, a decrease of the *b* value for the yellow paper and an increase of the *b* value for the blue printed paper are noticeable. These variations suggest that plasma treatment leads to a brighter paper and lower color values, thus to higher ink removal than without plasma treatment in those conditions. These differences of color are supported by image analysis performed using ImageJ software by converting the images to 8-bit grayscale images (0 corresponding to black and 250 to white) and by plotting the distribution of grey levels for the same amount of measured pixels (*i.e.* same surface area of the picture) (Fig. S2). The image analysis is performed on treated and non-treated areas immersed and not immersed and the same conclusions as the colorimeter and reflectance measurements can be drawn.

**Figure 4:**
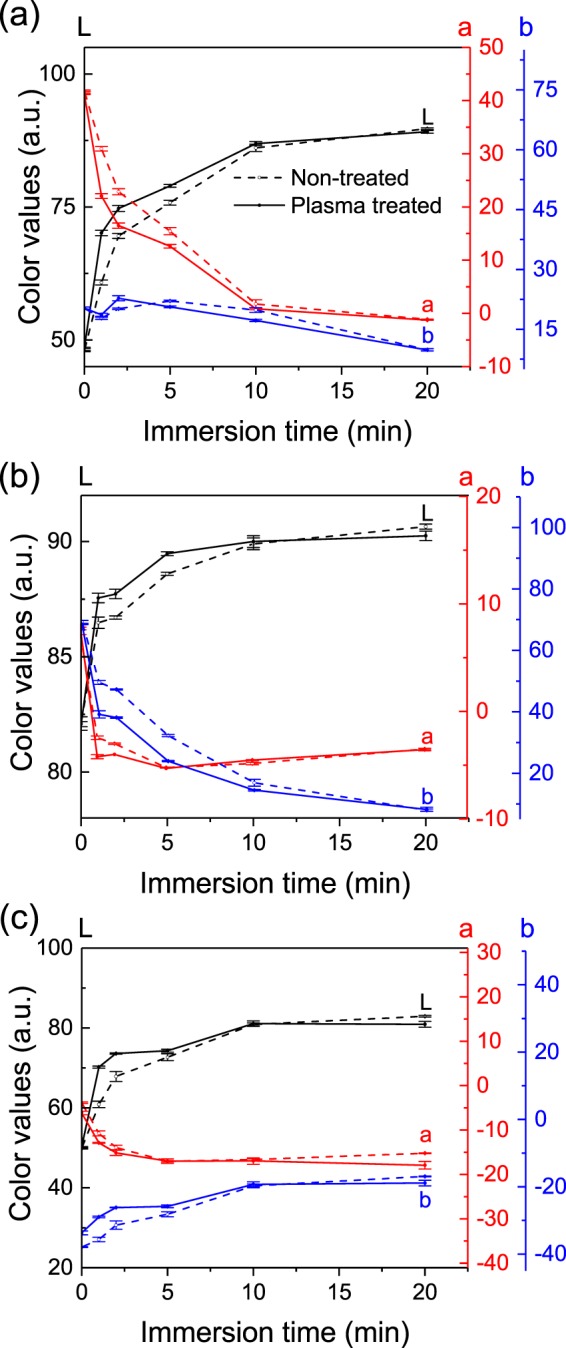
Effect of the immersion time in water on the color values of plasma treated (solid lines) and non-treated (dash lines) areas of (**a**) red, (**b**) yellow and (**c**) blue printed papers.

In order to assess in detail the effect of the plasma in the de-inking of paper, the immersion time in water and the plasma treatment duration are tuned. The effect of the immersion time in water on the *L-a-b* color values changes of 3 minutes plasma-treated and non-treated areas of printed papers are reported in Fig. 4. Papers are immersed for 1, 2, 5, 10 and 20 minutes in water under stirring and then dried. For each color, we can notice, in the 0 to 5 minutes immersion time range, a large color difference between the plasma-treated and non-treated areas. This color difference tends to decrease with the immersion time as the ink is progressively removed from the paper by the water, and from 10 minutes immersion, the printed papers reach similar color values irrespectively of the treatment. Even though similar values are reached for long immersion, the plasma treatment is clearly accelerating the initial stage of the de-inking process.

**Figure 5:**
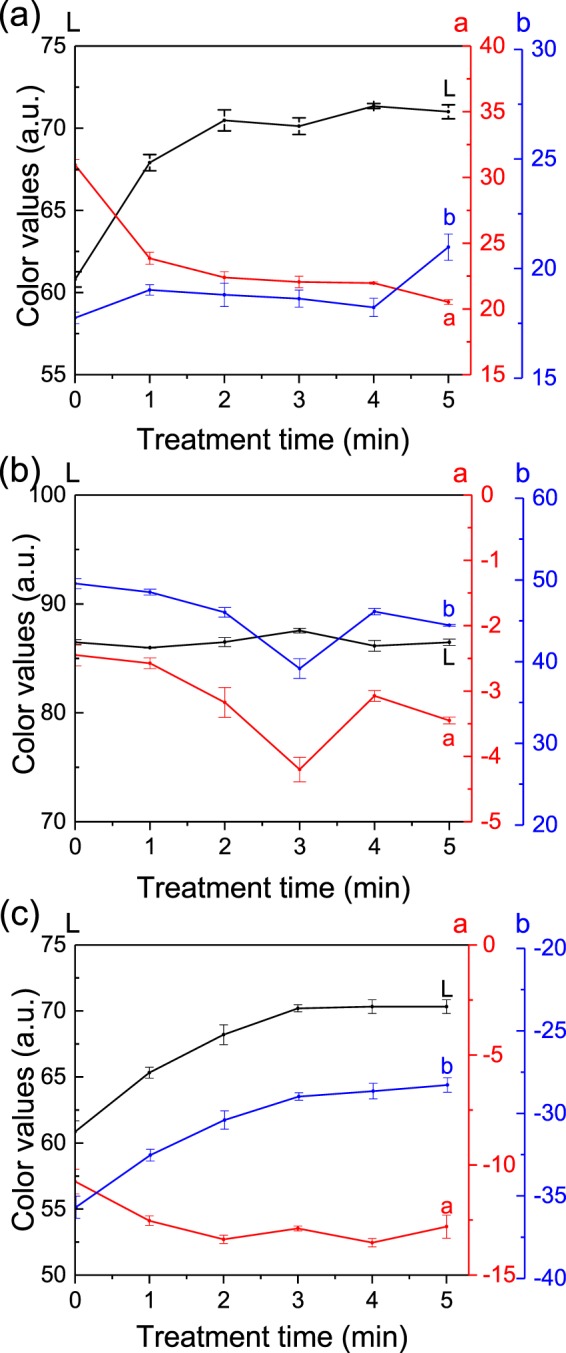
Effect of the plasma treatment time on the color values of (**a**) red, (**b**) yellow and (**c**) blue printed papers immersed 1 minute in water.

Figure 5 shows the *L-a-b* color values measurements of plasma-treated areas, for 0, 1, 2, 3, 4, and 5 minutes, of printed papers immersed 1 minute in water and dried. For the red paper, the increase of plasma treatment time leads to a clear increase of the *L* value and to the decrease of the *a* value, both values reaching a plateau after 2 minutes of treatment time. In the case of the yellow printed paper the *L* value increase slightly to reach a maximum at 3 minutes of treatment time. The *b* value of yellow printed paper is decreasing with an increased treatment time and is reaching a minimum at 3 minutes treatment time. In the case of the blue paper, the *L* value as well as the *b* value are increasing with the treatment time, both reaching a plateau at 3 minutes of treatment time. A 3 minutes plasma treatment time is then used for the rest of the study.

### Role of plasma in the de-inking mechanism

**Figure 6:**
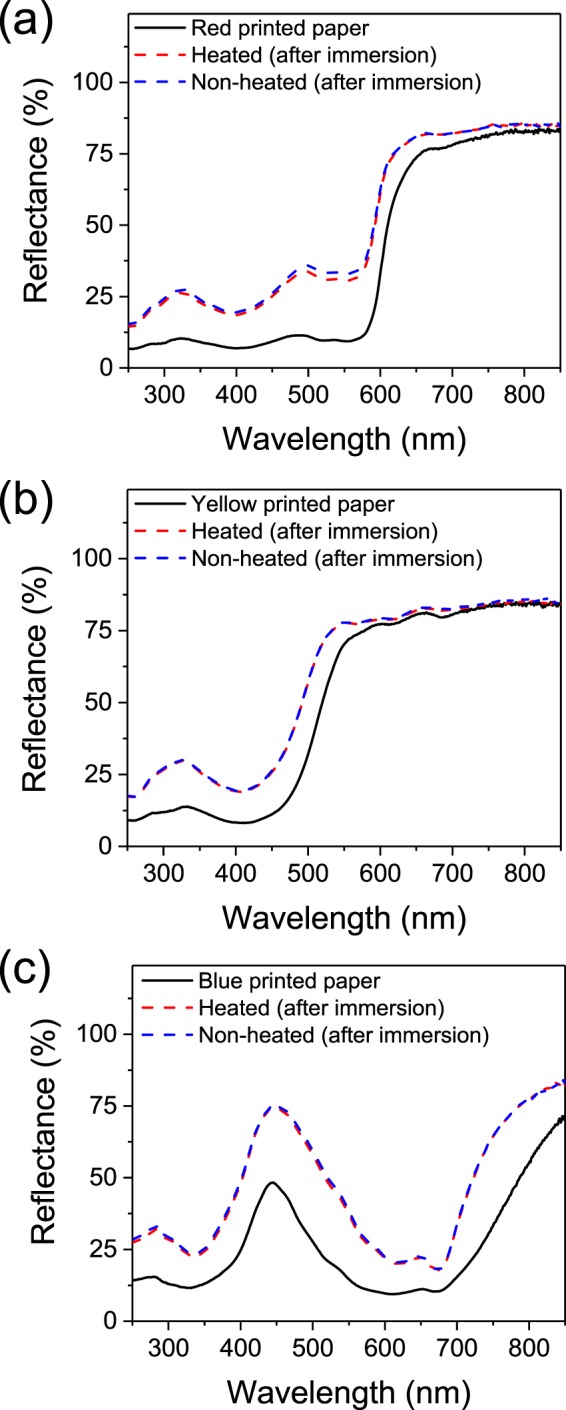
Reflectance measurements of non-heated and heated (**a**) red, (**b**) yellow and (**c**) blue printed papers (non-plasma-treated) after immersion in water. For each color, a reference printed paper reflectance spectrum before immersion is reported (black solid line).

**Figure 7:**
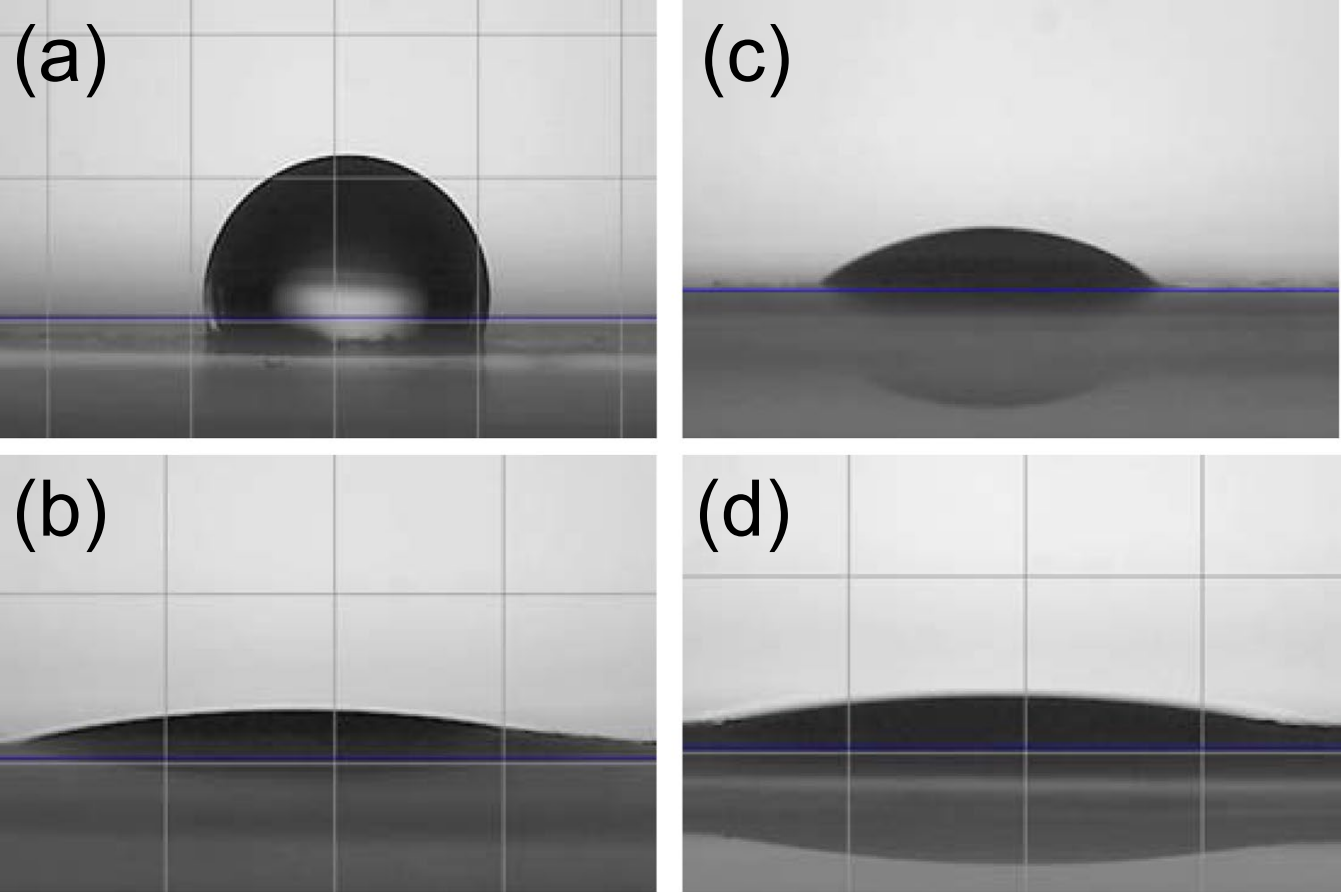
Water contact angle measurements on (**a**) non-printed white paper, (**b**) plasma-treated white paper, (**c**) blue printed paper, (**d**) plasma-treated blue printed paper (pictures taken about 1 second after the drop touches the surface).

During plasma treatment the surface temperature of the paper is measured to reach up to 60 °C after several minutes of treatment. In order to understand the part of the plasma self-heating effect in the de-inking process, printed papers are heated during 3 minutes at 60 °C and then immersed in water during 1 minute and their reflectance are measured (Fig. 6). As reported in Fig. 6, upon immersion both heated or non-heated papers shows an increase in reflectance compared to reference printed papers not immersed in water, showing a removal of the ink. However when comparing heated or non-heated paper the reflectance values are almost similar. The part of the plasma self-heating in the de-inking process seems to be negligible. The enhanced de-inking effect observed upon plasma treatment in the first part of the article is then likely to come from the plasma effect itself. In order to investigate the surface changes induced by plasma treatment the water contact angle is measured. As depicted in Fig. 7a, a non-printed non-plasma-treated paper surface is hydrophobic due to the presence of hydrophobic compounds introduced during paper making^10^. A water drop deposited on the surface stays on the surface of the paper and is absorbed very slowly (Supplementary Material, Video S1). Surface plasma treatment leads to the increase of hydrophilicity of the non-printed paper (Fig. 7b), a deposited drop is directly wetting totally the surface and is absorbed rapidly by the paper (Supplementary Material, Video S2). The blue printed paper surface (Fig. 7c), on the other hand, is hydrophilic, surely due to the hydrophilic properties of the deposited ink particles (Supplementary Material, Video S3)^11,12^. Even though the effect of exposure to plasma for blue printed paper is less clear than for white non-printed paper, a slight increase of hydrophilicity seems to be observable (Fig. 7d) and the water droplet is absorbed faster (Supplementary Material, Video S4).

**Figure 8:**
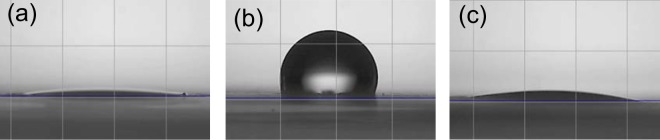
Water contact angle measurements performed on the backside of (**a**) plasma-treated white paper (**b**) blue printed paper (**c**) plasma-treated blue printed paper (picture taken about 1 second after the drop touches the paper surface).

In order to have more information on the modification induced by plasma treatment we measured the water contact angle on the backside of the samples (Fig. 8). For the white non-printed paper (Fig. 8a), even if only the front side is treated by plasma, the backside is also hydrophilic and the water is absorbed directly (Supplementary Material, Video S5). This is surely due to the depth penetration of the plasma species^12,14^, able to lead to surface changes even on the backside through the pores of the paper. The backside of a blue printed paper (Fig. 8b), not plasma treated, remains hydrophobic. However after few seconds, the deposited droplet is absorbed by the paper (Supplementary Material, Video S6). The penetration of some ink particles deep in the paper might be the reason of the faster absorption of water compared to non-printed white paper.

As reported for the plasma-treated white paper backside, the plasma-treated blue printed paper backside (Fig. 8c) is hydrophilic and absorbs water fast (Supplementary Material, Video S7). The increase of hydrophilicity and the rapid absorption of water by both sides of the paper might be the reason of the observed fast de-inking. The fibers are likely to swell faster and ink particles from inside the paper can be removed faster compared to non-plasma-treated paper, presenting hydrophobic properties with a slower water absorption. Indeed, Gaiolas *et al*. suggested through fiber width and length measurements that plasma-treated paper fibers have a higher ability to swell^10^. A difference of absorption speed and swelling could explain the delay observed in Fig. 4 to reach the same de-inking efficiency, *i*.*e*. about 10 minutes, for various immersion time of treated and non-treated printed paper. It is worth noting that treated paper surface remains highly hydrophilic even after 2 weeks stored at air.

**Figure 9:**
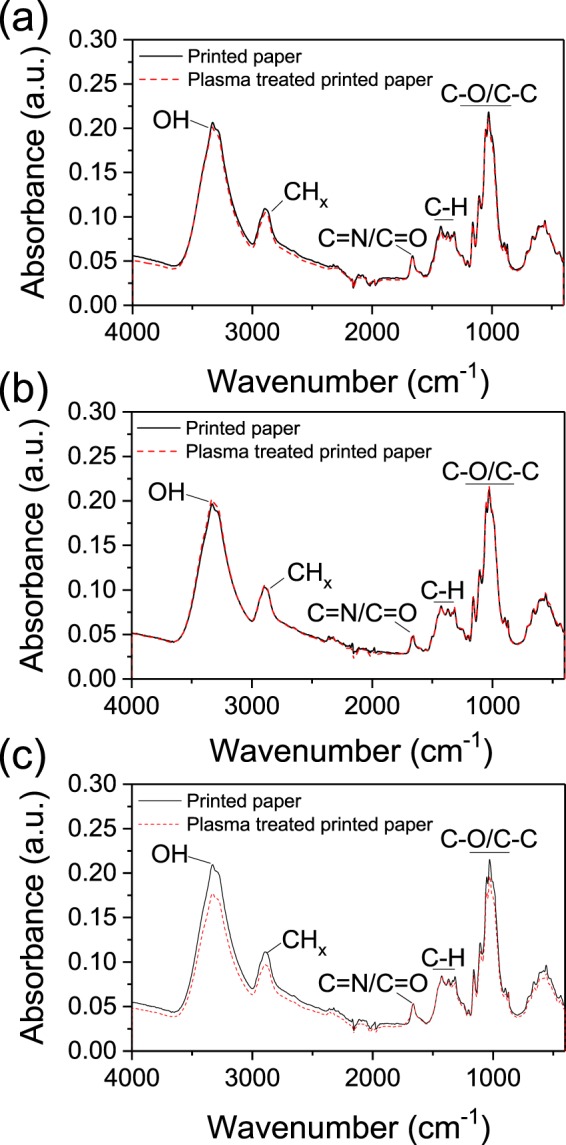
FTIR spectra for (**a**) red, (**b**) yellow and (**c**) blue printed paper, non-plasma-treated and plasma-treated.

To find out the effect of plasma on the paper chemistry, FTIR analyses are performed. Non-treated and plasma treated papers are compared in Fig. 9. Due to the penetration depth of infrared radiations, for all samples, the observed bonds are belonging mainly to the cellulose composing the paper. Indeed, characteristics bonds of cellulose, e.g. OH, C-O, C-C, CH are observed as reported in the literature^15,16^. The presence of the peak at 1663 cm^−1^, which is of very low intensity and broaden in the spectrum of the white paper (Fig. S3), might be assigned to C=N bonds present in the inks compounds^17^. This component is possibly overlapping with other bonds such as C=O or NH. With or without plasma treatment, no significant changes are noticeable, suggesting that the low temperature plasma treatment is likely to not lead to the degradation of the paper quality.

**Figure 10:**
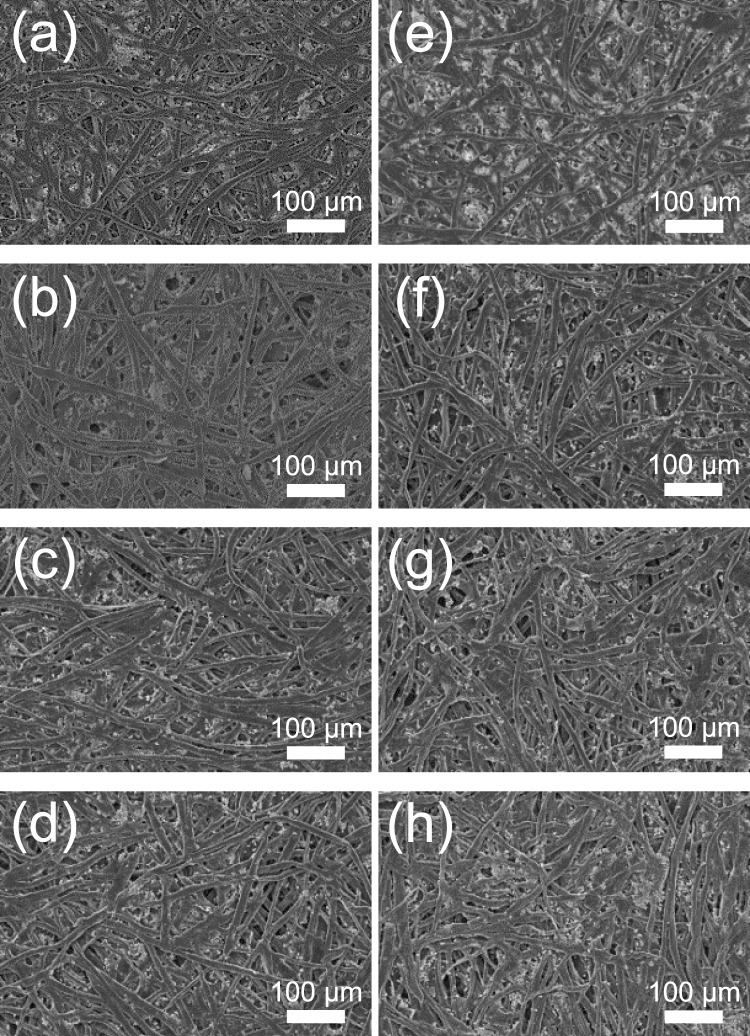
SEM micrographs of (**a**–**d**) non-plasma-treated: non-printed (**a**), red (**b**), yellow (**c**) and blue (**d**) printed papers and (**e**–**h**) plasma-treated: non-printed (**e**), red (**f**), yellow (**g**) and blue (**h**) printed papers.

The surface of non-treated printed paper and plasma-treated printed paper are observed by SEM (Fig. 10). Comparing the paper fibers surface before (Fig. 10a–d) and after plasma processing (Fig. 10e–h), for white paper or inkjet-printed paper, no differences can be noticed.

The fibers do not present any apparent increase of surface roughness, decrease of the fiber diameter or damage in fiber structure. Compared to some other works observing surface modification of the paper fibers upon plasma treatment, such as increase of surface roughness^11,12^, our study suggest that the performed low power plasma treatment does not damage the paper morphology. It is worth noting that Gaiolas *et al*., treating paper with a low pressure plasma, also did not observe any morphological damage on the plasma-treated paper fibers^10^. As surface wetting is dependent of the surface morphology and chemistry, the absence of apparent increase of roughness suggests that the increase of surface hydrophilicity is mainly due to a change of surface chemistry. Indeed, the highly reactive species present in the plasma such as oxygen-containing species coming from the surrounding environment of the plasma source leads to the oxidation of the surface of the fibers and the formation of groups such as OH or COOH^12,18^. Tensile tests are also performed to assess that the paper mechanical properties are not affected by the plasma treatment. Since the chemical and physical properties of the paper are not significantly changed by plasma treatment, the tensile strength of treated and non-treated papers would also remain similar. From the tensile tests, the forces at break for non-treated white paper (4.65 ± 0.9 N) and plasma treated white paper (4.77 ± 0.3 N) are the same. This indirectly suggests that no damage seems to be induced by plasma treatment.

## Conclusion

The treatment of inkjet-printed papers by a helium plasma is reported to accelerate the de-inking of papers. By plasma treating the surface of yellow, red or blue printed papers and subsequently immersing them in water, a faster ink removal is observed in the plasma-treated areas compared to non-treated areas of the papers. As swelling of paper fibers favors the ink particles detachment, the increase of surface hydrophilicity induced by the plasma treatment is believed to play an important role in the de-inking enhancement. Indeed, interestingly, upon plasma treatment, not only the front side of the paper exposed directly to the plasma becomes highly hydrophilic, but also the backside not directly exposed. The plasma species are likely to penetrate deeply through the paper fibers. The increased wetting of both sides of the paper leads to the fast absorption of water compared to non-plasma-treated paper and is believed to enhance the fiber swelling of the paper and accelerate the ink removal. It is worth noting that for long immersion times in water, non-treated or plasma-treated areas of the paper reach the same de-inking efficiency. This could be explained by the fact that the non-treated printed paper backside absorbs water slowly and thus a longer time is needed to reach the same fiber swelling and ink removal amount as the plasma-treated paper. Through FTIR analyses, SEM observations and tensile tests, the paper quality seems to be retained, making this method promising for further paper recycling. Plasma treatment appears to be interesting to assist current ink removal processes and the overall recycling process. In addition to the ink removal, plasma treatment is reported in the literature to decrease the disintegration time of paper thanks to the increase of surface wetting, reducing time and energy spent in the recycling process. We believe that plasma treatment may lead to the decrease of the use of chemicals and generated wastes during ink removal processes and paper recycling.

## Supplementary information

Supplementary info

Video S1

Video S2

Video S3

Video S4

Video S5

Video S6

Video S7

## Data Availability

The authors declare that the data supporting the findings of this work are available within the paper. All additional raw and derived data that support the plots within the paper and other findings of this work are available from the corresponding author upon reasonable request.
