# Supplementary material for: Improved de-inking of inkjet-printed paper using environmentally friendly atmospheric pressure low temperature plasma for paper recycling
Source: Sci Rep. 2019 Oct 1;9:14046. doi: 10.1038/s41598-019-50495-4 (PMC6773741; doi:10.1038/s41598-019-50495-4)
Supplement: Supplementary file 1 — Supplementary info [file 41598_2019_50495_MOESM1_ESM.docx]

Supplementary material

**Improved de-inking of inkjet-printed paper using environmentally friendly atmospheric pressure low temperature plasma for paper recycling**

Rodolphe Mauchauffé^1^^[[1]](#footnote-1)^ , Seung Jun Lee^1^*, Isaac Han^1^*, Sang Hyeong Kim^2^, Se Youn Moon^1^^[[2]](#footnote-2)^

*^1^ Department of Quantum System Engineering, Chonbuk National University, 567 Baekje-daero, Deokjin-gu, Jeonju, Jeollabuk-do, 54896, Republic of Korea*

*^2^ Altoran Research Institute, 10-41, Bokyongbuk-ro 17, Yuseong-gu, Daejeon-city, 34160, Republic of Korea*

**
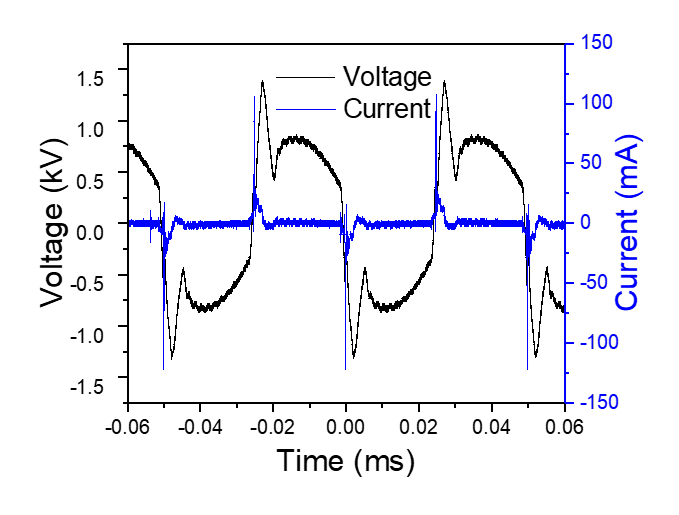
**

**Figure S1:** Discharge voltage and current measurement.

**Figure S2:** Digital analysis of non-treated and plasma-treated red, yellow and blue printed papers before and after immersion in water.


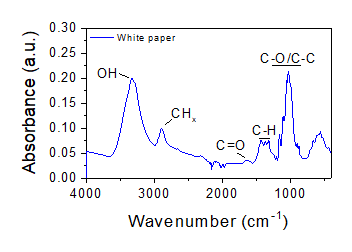


**Figure S3:** FTIR spectrum of reference white paper.

**Supporting videos**

Video S1: non-printed white paper with 2× speed

Video S2: plasma-treated white paper

Video S3: blue-printed paper

Video S4: plasma-treated blue-printed paper

Video S5: plasma-treated white paper backside

Video S6: Blue-printed paper backside

Video S7: plasma-treated blue-printed paper backside

1. These authors contributed equally to this work. [↑](#footnote-ref-1)
2. Authors to whom correspondence should be addressed. E-mail: symoon@jbnu.ac.kr [↑](#footnote-ref-2)
